# EMSY enhances glycolysis in ovarian cancer cells

**DOI:** 10.1186/s40001-025-03133-x

**Published:** 2025-09-29

**Authors:** Shaosheng Wang, Na Xie, Ping Zha, Yingming Li, Minghui Wei, Ji Bai, Xiaohong Zhao

**Affiliations:** 1 Blood Transfusion Department, Haikou People’s Hospital, Haikou, 570208 Hainan China; 2 Department of Pathology, The affiliated hospital of Hainan Medical University, Haikou, 571101 Hainan China; 3 Women Health Care Department, Hainan Women and Children Medical Center, Haikou, 570312 Hainan China

**Keywords:** Ovarian cancer, EMSY, β-catenin, LDHA

## Abstract

EMSY is critical in the progression of ovarian cancer; nevertheless, its impact on tumor metabolism is not widely known. This study showed ovarian cancer cells overexpressing EMSY produce more lactate and have upregulated LDHA expression; the promoting effect of EMSY on the malignant phenotype of these cells is eliminated when LDHA expression is knocked down or glycolysis is inhibited in ovarian cancer cells. Mechanistic studies revealed that EMSY interacts with β-catenin, and the knockdown of EMSY inhibits the transcriptional activation of LDHA by β-catenin. In summary, this study identifies EMSY’s metabolic regulatory role in ovarian cancer cells and offers treatment of ovarian cancer with a new therapeutic target.

**Supplementary Information:**

The online version contains supplementary material available at 10.1186/s40001-025-03133-x.

## Introduction

Ovarian cancer, a common cancer type affecting the female reproductive system, originates from the ovary’s epithelial, germ, or stromal cells [1, 2]. Due to its insidious early symptoms, the majority get diagnosed when it is already at the advanced stage, leading to poor prognosis and the highest mortality rate among gynecological malignancies [3, 4]. Urgent priorities include the investigation of the pathogenesis of ovarian cancer and the identification of therapeutic targets.

EMSY (a transcriptional regulator interacting with BRCA2) is located on chromosome 11 and has 21 exons [5, 6]. The EMSY protein, consisting of 1,068 amino acids, contains a conserved domain at the N-terminus and a BRCA2-binding domain at the C-terminus [7]. EMSY is a gene closely associated with DNA damage repair [7]. Initially identified as functionally aberrant in breast and ovarian cancers [7, 8], it promotes tumorigenesis by disrupting the BRCA pathway and serves as a key driver of genomic instability [9]. The beta-catenin/TCF signaling pathway is activated by EMSY in ovarian cancer cells [10]. However, the downstream molecular biological events are still unclear.

LDHA (Lactate Dehydrogenase A), a critical isoform of the lactate dehydrogenase (LDH) family, catalyzes the conversion of pyruvate to lactate, the final step of glycolysis [11]. LDHA is vital in regulating metabolic reprogramming in tumors, e.g., the Warburg effect [11, 12]. The Warburg effect promotes tumor invasion, metastasis, and drug resistance [13]. Multiple oncogenic signaling pathways have been identified as regulators of LDHA expression. Hypoxia induces HIF-1α to upregulate LDHA, forming a positive feedback loop [14]. Wild-type p53 suppresses LDHA, while mutant p53 may enhance its activity [15]. Studies in lung cancer cells also report beta-catenin/TCF complex binding sites in the LDHA promoter, directly elevating mRNA levels [16]. However, the regulatory mechanisms and functions of LDHA in ovarian cancer remain poorly understood.

This study examined how EMSY expression regulates glycolysis in tumor cells and its interaction with the beta-catenin/TCF signaling pathway in modulating LDHA expression levels.

## Materials and methods

### Cell culture

The human ovarian cancer cell lines OVCA429 (RRID:CVCL_3936), OVCA433 (RRID:CVCL_0475), and HEK293T (RRID:CVCL_0063) were obtained from the Chinese Academy of Sciences Cell Bank in Shanghai, China. HEK293T and ovarian cancer cells were cultured in DMEM enriched with 10% fetal bovine serum, 100 mg/mL streptomycin, and 100 U/mL penicillin to enhance cell viability and prevent contamination. In a humidified incubator with 5% CO_2_, all cells were cultured at 37 °C. The Lipofectamine 8000 reagent was used for cell transfection following the manufacturer’s instructions.

### Clinical samples

The human female ovarian cancer tissues from Hainan Provincial Maternal and Child Health Hospital were collected. All tissue samples were following patients signing informed consent forms. A total of 6 pairs of samples were used in this study. The Hainan Provincial Maternal and Child Health Hospital’s Ethics Committee approved this study.

### qPCR

TRIzol reagent (Invitrogen) was utilized to extract total RNA, and 1 µg of the isolated RNA was subsequently converted into cDNA using the PrimeScript™ RT Kit (Takara) following the manufacturer’s guidelines. A CFX96 Real-Time PCR Detection System (Bio-Rad, Richmond, CA, USA) performed quantitative real-time PCR with the SYBR Green Kit. Using β-actin as the reference gene, the 2^− ΔΔCt^ method was used to ascertain the target gene’s relative expression.

### IHC

After obtaining informed consent from the patients, ovarian cancer samples and adjacent tissues were taken from Hainan Provincial Maternal and Child Health Hospital. Hospital Institutional Ethics Committee ethical permission was acquired. Before antigen retrieval in EDTA solution, tissue slices were deparaffinized and rehydrated for 30 min at 100 °C. After a natural cooling time to room temperature, a blocking agent was incubated for 15 min to inhibit endogenous peroxidase activity. The tissue slices were then treated with LDHA (Proteintech, 19,987–1-AP, 1:100) and EMSY (Proteintech, 26,212–1-AP, 1:100) primary antibodies over the course of the night at 4 °C. The tissue sections were washed with PBS and incubated with secondary antibodies for 1 h at room temperature. To observe immunohistochemical signals, 3,3-diaminobenzidine (DAB) was used. The hematoxylin counterstain was applied to every tissue section.

### Western blot

The cells were lysed on ice with RIPA lysis buffer containing protease and phosphatase inhibitors, following two washes with PBS. The BCA protein assay kit was employed to ascertain the protein concentration in the supernatant derived from the centrifuged cell lysate. SDS–PAGE gel electrophoresis was used to separate proteins in equal amounts, and the resultant proteins were subsequently transferred onto PVDF membranes. The membranes were blocked with BSA and incubated with various primary antibodies specific to the target proteins at 4 °C for the entire night. These included anti-Tubulin (Santa Cruz Biotechnology, sc-5286, 1:4000), anti-GAPDH (Proteintech, 60,004–1-lg, 1:1000), anti-EMSY (Proteintech, 26,212–1-AP, 1:100), anti-HA (Proteintech, 81,290–1-RR, 1:1000), anti-Flag (Proteintech, 66,008–4-lg, 1:1000), anti-β-catenin (Proteintech, 51,067–2-AP, 1:1000), and anti-LDHA (Proteintech, 21,799–1-AP, 1:100) antibodies. This incubation step facilitated specific binding of the antibodies to their respective protein targets in preparation for downstream analysis. After adding HRP-conjugated secondary antibodies, the membranes were incubated for 1–2 h. Image Lab software was utilized for investigation after the immunosignals were found employing a chemiluminescent substrate (Millipore, WBKLS0050).

### CCK8 assay

A controlled incubator with 5% CO₂ was used to maintain the cells at 37 °C after they were plated in 96-well plates at a density of 1 × 10^3^ cells per well. Once the culture media was switched to a new medium supplemented with 10% CCK8 reagent, the cells were incubated in the incubator for 2 more Hours. The experimental steps were carried out daily, and absorbance was quantified at 450 nm.

### Determination of intracellular lactate

After culturing ovarian cancer cells for 24 h, samples of cell lysates or culture supernatants were collected. Lactate levels were then quantified by measuring absorbance at 450 nm using the Lactate Assay Kit-WST (Dojindo), following the protocol provided by the manufacturer.

### Soft agar assay

Cell confluence was around 50% at the following day after seeding in 6 cm dishes. After being detached, the cells were made into a suspension. 1.25% agar (40%), 2 × RPMI 1640 (Basal Medium Eagle, 40%), and FBS (20%) were used to produce the lower layer. Each well was then filled with 400 μL of the Mixture, which was then left to solidify at 37 °C. The cell suspension was Mixed with the prepared upper layer. Each well was then filled with 400 μL of this Mixture, which contained roughly 1× 10^3^ cells. The plates were then incubated in a humidified incubator for 10–14 days at 37 °C with 5% CO₂. The top layer Mixture comprised 25% fetal bovine serum (FBS), 37.5% 2 × RPMI 1640 medium, 37.5% 1% agar, and 0.8% 2 mM L-glutamine. Colonies were counted under a microscope by examining five randomly chosen fields.

### Reporter assay

Cells were seeded in a 12-well plate and reached the 50% confluence. Then, they were co-transfected with Renilla luciferase (0.02 μg), 0.05 μg of reporter plasmid, and 0.1 μg of expression vector. The reporter’s activity was measured 24 h after transfection using the Dual-Luciferase Reporter Assay System (Beyotime, RG088M). The experiment was performed three times.

### Immunoprecipitation assay

Using lipofectamine 8000, transfection was performed to determine how exogenously expressed proteins interacted. Cells were lysed 48 h after transfection using IP lysis buffer that contained 1% NP-40, 50 mM Tris–HCl (pH 8.0), 150 mM NaCl, and protease and phosphatase inhibitors. The resulting supernatant was incubated overnight at 4 °C with HA beads (Thermo Fisher Scientific, 88,836) and Flag beads (Sigma, A2220). The beads were incubated with 1 × loading buffer and heated for 5 min the next day after being cleaned three times with a wash buffer that contained 50 mM Tris–HCl (pH 8.0), 150 mM NaCl, and 1% NP-40. The supernatant was subjected to a western blot analysis.

IP lysis buffer containing protease and phosphatase inhibitors was used to lyse the cells to find relationships between endogenously generated proteins. After the supernatant was collected, it was incubated overnight at 4 °C with 1 μg of primary antibody. It was then incubated for 4 h at 4 °C with 40 μL of Protein A/G beads (bimake.com, B23202). After three washes with wash buffer, the beads were heated for 5 min using 1 × loading buffer. The supernatant was used for the Western blot.

### Statistical analyses

SPSS version 23.0 and GraphPad Prism version 8.0.2 (GraphPad Software, La Jolla, CA, USA) were used for the statistical analyses. A statistical test that was employed to analyze the data was the two-tailed Student’s *t* test. The threshold value of *P* was < 0.05.

## Results

### Overexpression of EMSY upregulates LDHA and promotes acidification of the culture medium

**Fig. 1:**
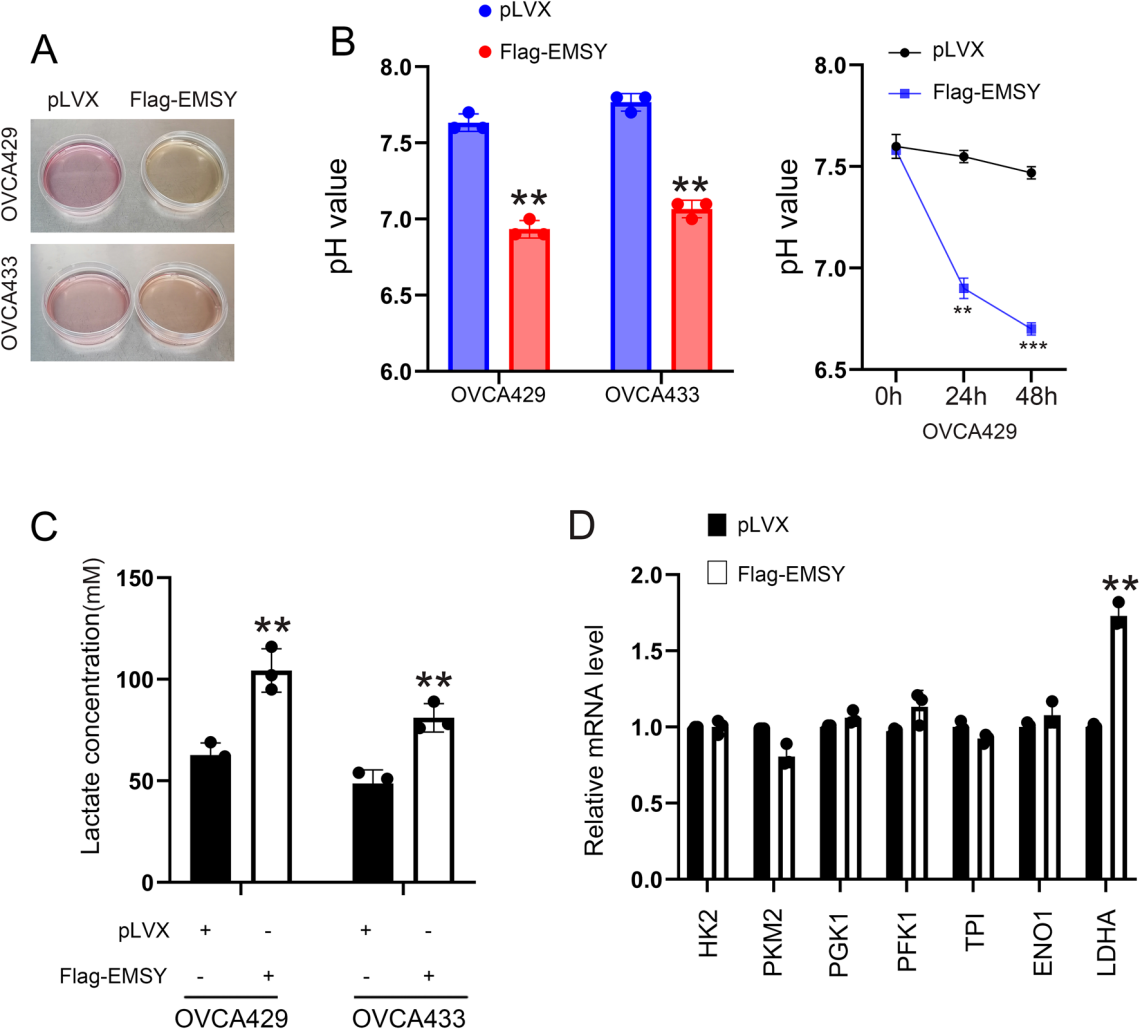
Overexpression of EMSY promotes lactate production. **A** Effect of EMSY overexpression on culture medium color in OVCA429 and OVCA433 cells. **B** Left: effect of EMSY overexpression on pH levels of culture medium in OVCA429 and OVCA433 cells. Right: the pH value in a time window. **C** Effect of EMSY overexpression on lactate levels of culture medium in OVCA429 and OVCA433 cells. **D** Effect of EMSY overexpression on the expression of glycolysis-related metabolic enzymes in OVCA429 cells. **P < 0.01; ***P < 0.001

In the cell culture, we noted that the supernatants of the culture medium from ovarian cancer cells OVCA429 and OVCA433, which overexpress exogenous EMSY (Flag-EMSY), exhibited a more rapid yellowing (Fig. 1A). This suggested that EMSY-overexpressing cells might promote the medium acidification. To validate this hypothesis, we first measured the pH of the culture medium. Results showed a decrease in pH following EMSY overexpression (Fig. 1B). Consistent with these observations, lactic acid levels in the medium were elevated upon EMSY overexpression (Fig. 1C). We analyzed the mRNA expression of glycolytic enzymes using qPCR to investigate the underlying mechanism for the increased lactic acid production. The mRNA expression of LDHA was markedly elevated in cells overexpressing EMSY among the metabolic enzymes associated with glycolysis (Fig. 1D). Consequently, we focused on elucidating how EMSY regulates LDHA expression and the role of LDHA in mediating EMSY-driven oncogenic effects.

### Overexpression of EMSY upregulates LDHA protein levels

**Fig. 2:**
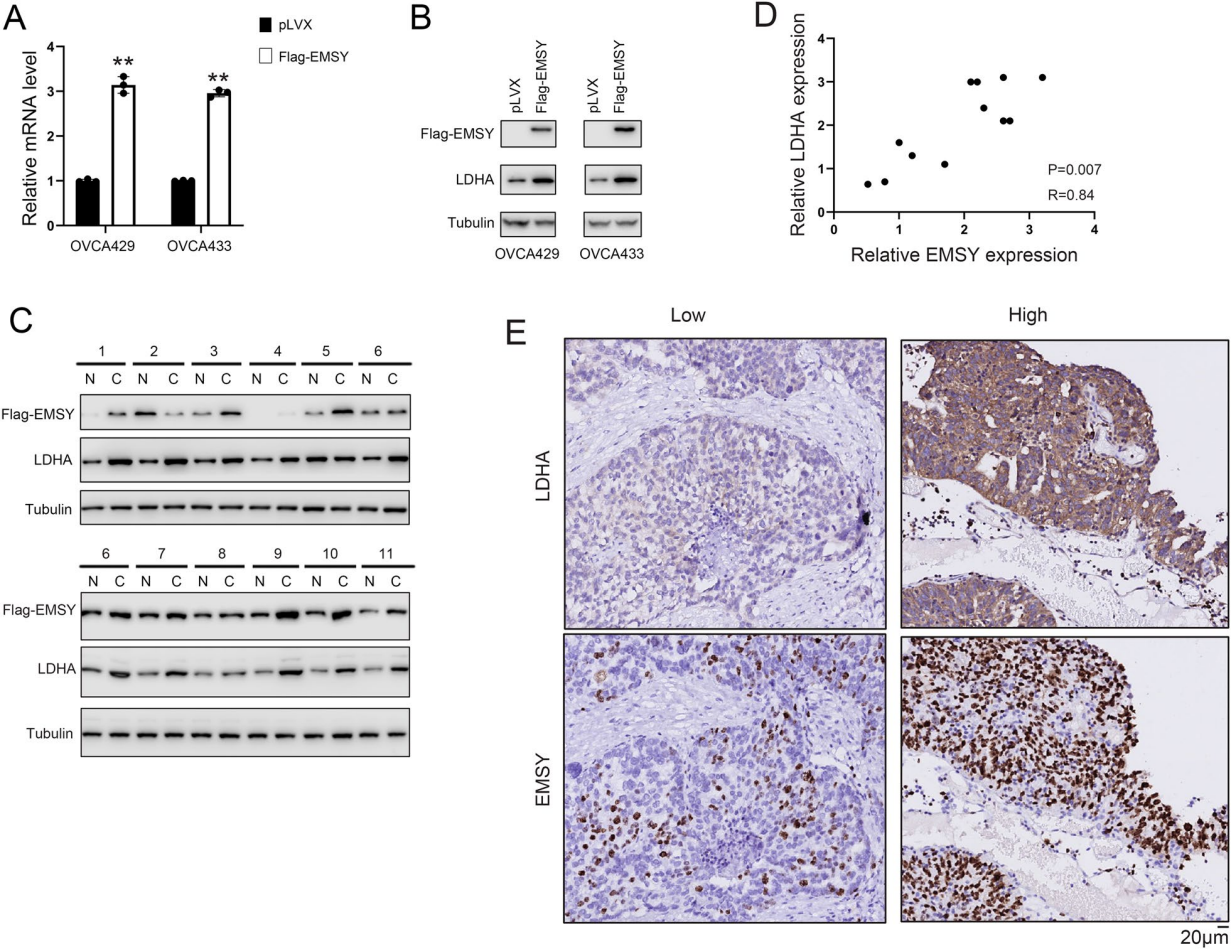
Overexpression of EMSY upregulates LDHA expression. **A** Effect of EMSY overexpression on LDHA mRNA levels detected by qPCR. **B** Effect of EMSY overexpression on LDHA protein levels detected by Western blot. **C** Protein levels of EMSY and LDHA in ovarian cancer tissues and adjacent non-cancerous tissues detected by Western blot. **D** Correlation analysis of the EMSY and LDHA protein level. **E** EMSY and LDHA protein levels in ovarian cancer tissues and adjacent non-cancerous tissues were detected by immunohistochemical (IHC) staining. “High”, the tissues with higher expression of EMSY; “low”, the tissues with lower expression of EMSY. **P < 0.01

We first employed qPCR and Western blot to examine the effects of EMSY overexpression on LDHA expression at both protein and mRNA levels. The data showed that increasing EMSY levels upregulated LDHA mRNA and protein (Fig. 2A, B). Furthermore, in ovarian cancer tissues, we observed upregulated expression of both EMSY and LDHA in tumor tissues, and the expression of LDHA positively correlated with that of EMSY (Fig. 2C, D). This phenomenon was further confirmed through immunohistochemical staining analysis (Fig. 2E).

### Knockdown of EMSY downregulates LDHA expression

**Fig. 3:**
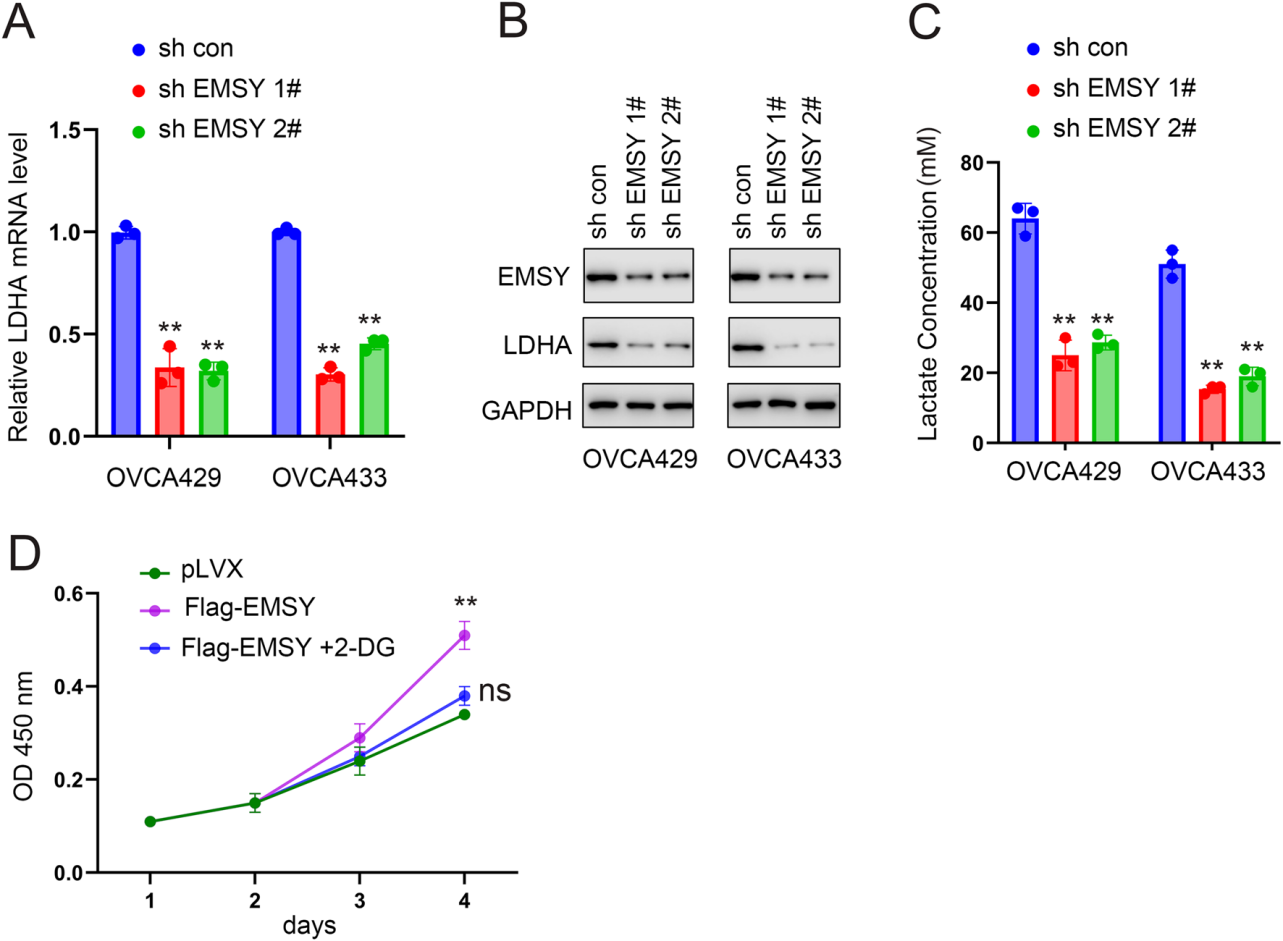
Downregulation of EMSY inhibits LDHA expression. **A** Effect of EMSY knockdown on LDHA mRNA levels detected by qPCR. **B** Effect of EMSY knockdown on LDHA protein levels detected by Western blot. **C** Effect of EMSY knockdown on lactate content. **D** Effect of the glycolysis inhibitor 2-DG on cell proliferation detected by CCK8 assay. **P < 0.01

The effects of EMSY knockdown on LDHA expression were first examined using qPCR and Western blot. The studies demonstrated that OVCA429 and OVCA433 cells, cells with ovarian cancer, had lower levels of LDHA mRNA and protein when EMSY expression was knocked down (Fig. 3A, B). Consistent with this, EMSY knockdown also suppressed lactate levels in these cells (Fig. 3C). Previous studies found that EMSY overexpression promotes ovarian cancer cell growth. To investigate whether this pro-growth effect depends on glycolysis, we intervened with the glycolysis inhibitor 2-DG. The outcomes showed that 2-DG almost completely abolished the growth-promoting effects of EMSY overexpression in OVCA429 cells (Fig. 3D).

### EMSY promotes ovarian cancer cell growth in an LDHA-dependent manner

**Fig. 4:**
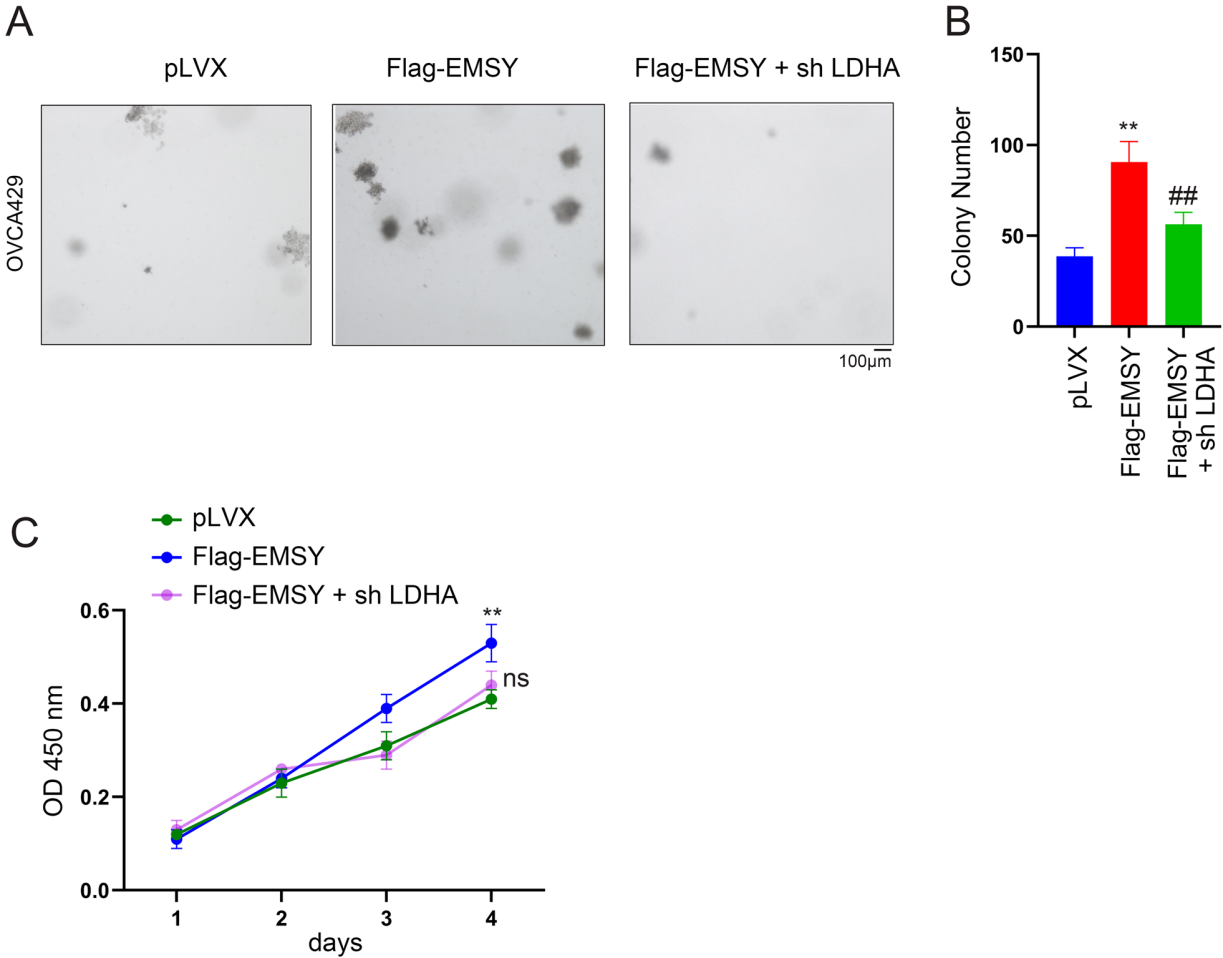
Knockdown of LDHA reverses the tumor-promoting effect of EMSY. **A** Effect of LDHA knockdown on anchorage-independent growth of ovarian cancer cells detected by soft agar colony formation assay. **B** Statistical analysis of colonies in (**A**). **C** Effect of LDHA knockdown on ovarian cancer cell proliferation detected by CCK8 assay. **P < 0.01; ^##^*P* < 0.01

To investigate whether EMSY’s oncogenic role in ovarian cancer relies on its regulation of LDHA, we performed LDHA knockdown in EMSY-overexpressing OVCA429 cells. Experimental results demonstrated that silencing LDHA in these cells nearly abolished the pro-growth effects of EMSY overexpression on anchorage-independent growth (Fig. 4A, B) and proliferation in liquid culture (Fig. 4C).

### β-Catenin regulates LDHA through EMSY-dependent mechanisms

**Fig. 5:**
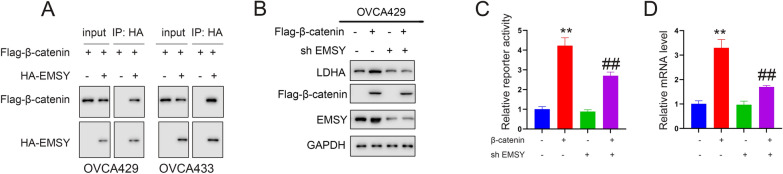
EMSY interacts with β-catenin to regulate LDHA expression. **A** Interaction between EMSY and β-catenin detected by co-immunoprecipitation (Co-IP). **B** Effect of EMSY knockdown and β-catenin overexpression on LDHA protein levels detected by Western blot. **C** Effect of EMSY knockdown on LDHA promoter activity detected by luciferase reporter assay. **D** Effect of EMSY knockdown on LDHA mRNA levels detected by qPCR. **P < 0.01; ^##^*P* < 0.01

Our previous work and this study demonstrated an interaction between β-catenin and EMSY (Fig. 5A). Given published evidence that β-catenin regulates LDHA expression, we investigated whether this regulatory axis requires EMSY. In ovarian cancer cells, β-catenin was overexpressed, followed by EMSY knockdown (Fig. 5B). Results showed that β-catenin overexpression activated the LDHA promoter and upregulated LDHA expression (Fig. 5C, D). Strikingly, EMSY silencing abolished β-catenin-mediated regulation of LDHA (Fig. 5C, D).

## Discussion

Metabolic reprogramming is a hallmark of cancer cells, distinguishing them from normal cells [17]. Even in the presence of oxygen, ovarian cancer cells, similar to other solid tumors, display the Warburg effect, preferring glycolysis to oxidative phosphorylation for energy production. This metabolic alteration facilitates rapid cell growth. Because ovarian cancer overexpresses important enzymes, such as pyruvate kinase M2 (PKM2), hexokinase 2 (HK2), and lactate dehydrogenase A (LDHA), there is an increase in glycolytic activity [18].

Glycolysis sustains tumor growth and chemoresistance through multifaceted mechanisms [19]. Glycolysis provides both ATP and critical biosynthetic precursors (e.g., NADPH and nucleotides), enabling rapid biomass production to fuel uncontrolled tumor proliferation [19]. Moreover, excess lactate accumulation acidifies the tumor microenvironment, activating cancer-associated fibroblasts (CAFs) [20]. This process promotes immune evasion by suppressing anti-tumor immune responses, such as T-cell cytotoxicity and infiltration [21]. In addition, enhanced glycolytic flux drives the synthesis of glutathione GSH, a key antioxidant molecule [22]. Elevated GSH levels mitigate platinum-based drug efficacy (e.g., cisplatin) by neutralizing reactive oxygen species ROS-induced oxidative stress, a primary mechanism of chemotherapy-induced cell death [22].

Ovarian cancer stem cells (CSCs) depend on glycolysis to maintain stemness. Ovarian CSCs exhibit suppressed mitochondrial function, favoring glycolysis over oxidative metabolism [23]. The HIF-1α/PDK1 axis mediates this metabolic shift [24]. Furthermore, Glycolysis-derived metabolites, such as pyruvate, directly influence epigenetic landscapes to sustain pluripotency [25]. Since glycolysis plays an essential role in the progression of ovarian cancer, this process needs to be strictly regulated.

Oncogene activation and tumor suppressor gene inactivation majorly impact tumor metabolic reprogramming [26]. EMSY is predominantly characterized by gene amplification (copy number gain) in a subset of ovarian cancers. Approximately 10–15% of ovarian cancers exhibit EMSY amplification, which may drive genomic instability by disrupting the homologous recombination repair (HRR) pathway [27]. The regulatory role of EMSY in tumor metabolism is still inadequately understood. In addition, previous studies have demonstrated that LDHA is a downstream target gene of β-catenin signaling [16]. This study reveals that EMSY and β-catenin cooperatively regulate LDHA expression, with 10–15% of ovarian cancers harboring β-catenin mutations [28]. These results imply that LDHA inhibitors might target malignancies exhibiting abnormal Wnt/β-catenin signaling pathway activation and EMSY amplification.

The regulatory effect of EMSY on lactate production might influence protein lactylation modification. Currently, it has been found that RAD51 and PFKP [29, 30], among others, undergo lactylation modification in ovarian cancer, and the lactylation modification of these proteins further enhances their resistance to platinum-based drugs in ovarian cancer. This might also explain, from another perspective, the molecular mechanism by which EMSY promotes the progression of ovarian cancer.

In conclusion, this study shows that EMSY plays a metabolic regulatory role in ovarian cancer cells, indicating that LDHA could be a therapy target for ovarian malignancies that have EMSY amplification.

## Supplementary Information

Additional file 1. Figure S1. Western blot primary data for Fig. 2. Figure S2. Western blot primary data for Fig. 3. Figure S3. Western blot primary data for Fig. 5.

## Data Availability

No datasets were generated or analysed during the current study.

## Abbreviations

EMSY: A transcriptional regulator interacting with BRCA2
LDHA: Lactate Dehydrogenase A
PFKP: Phosphofructokinase, platelet
